# To What Extent is FAIMS Beneficial in the Analysis of Proteins?

**DOI:** 10.1007/s13361-015-1326-4

**Published:** 2016-02-02

**Authors:** Helen J. Cooper

**Affiliations:** School of Biosciences, University of Birmingham, Edgbaston, Birmingham, B15 2TT UK

**Keywords:** FAIMS, DMS, Proteins, Proteomics, Peptides

## Abstract

High field asymmetric waveform ion mobility spectrometry (FAIMS), also known as differential ion mobility spectrometry, is emerging as a tool for biomolecular analysis. In this article, the benefits and limitations of FAIMS for protein analysis are discussed. The principles and mechanisms of FAIMS separation of ions are described, and the differences between FAIMS and conventional ion mobility spectrometry are detailed. Protein analysis is considered from both the top-down (intact proteins) and the bottom-up (proteolytic peptides) perspective. The roles of FAIMS in the analysis of complex mixtures of multiple intact proteins and in the analysis of multiple conformers of a single protein are assessed. Similarly, the application of FAIMS in proteomics and targeted analysis of peptides are considered.

**Graphical Abstract:**
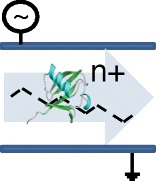
ᅟ

ᅟ

## Introduction

In contrast to mass spectrometry, ion mobility spectrometry (IMS) relies on the behavior of gas-phase ions in a gaseous medium. Collisions between the ion and the gas molecules or atoms (i.e., the ion mobility) dictate the movement of the ion. Ions in a drift gas that are subjected to an electric field will move in the direction of the field with velocity, *v*

1 $$ v=KE $$

where *K* is the ion mobility and E is the electric field strength. At low electric fields, ion mobility is independent of electric field strength; however, at high electric fields, ion mobility shows a nonlinear dependence on field strength [1]. The dependence of ion mobility on field strength can be described as

2 $$ {K}_h={K}_0\left[1+\alpha {\left[\frac{E}{N}\right]}^2+\beta {\left[\frac{E}{N}\right]}^4+\dots \right] $$

where *K*_*h*_ is the ion mobility at high field, *K*_*0*_ is the ion mobility at zero field strength, *E* is the electric field, *N* is the gas number density, and *α* and *β* are ion-specific coefficients. FAIMS takes advantage of these differences in ion mobility at high and low electric fields for the separation of ions at ambient pressure [2–4]. Although deriving from a similar physical basis, FAIMS is quite different from conventional ion mobility spectrometry, such as drift tube ion mobility spectrometry or traveling wave ion mobility spectrometry, both in the particular fundamental principles exploited in its implementation and (largely) in its applications, vide infra.

## What’s in a Name?

This technique, whereby ions at atmospheric pressure are separated on the basis of differences in their mobilities in high and low electric fields, goes by many sobriquets. It is variously known as *high field asymmetric waveform ion mobility spectrometry* [3, 4] (or simply *field asymmetric waveform ion mobility spectrometry* [5]), *differential ion mobility spectrometry*, *differential mobility spectrometry* [6], and the acronyms *FAIMS*, *DMS*, and *DIMS*. Early publications also used the terms *transverse field compensation ion mobility spectrometry* [2] and *field ion spectrometry* [7], although these have since fallen out of use. Some researchers have attempted to delineate the terms by assigning “FAIMS” to devices comprising cylindrical electrodes and “DMS” to those with planar electrodes. Others have applied c-FAIMS and p-FAIMS to distinguish between cylindrical and planar geometries [8]. The use of multiple names is a barrier to acceptance and adoption by the broader scientific community and this author urges researchers in the field to reach a consensus in the matter. In the current absence of consensus, this article will use the term ‘FAIMS’.

## Principles of FAIMS

**Fig. 1:**
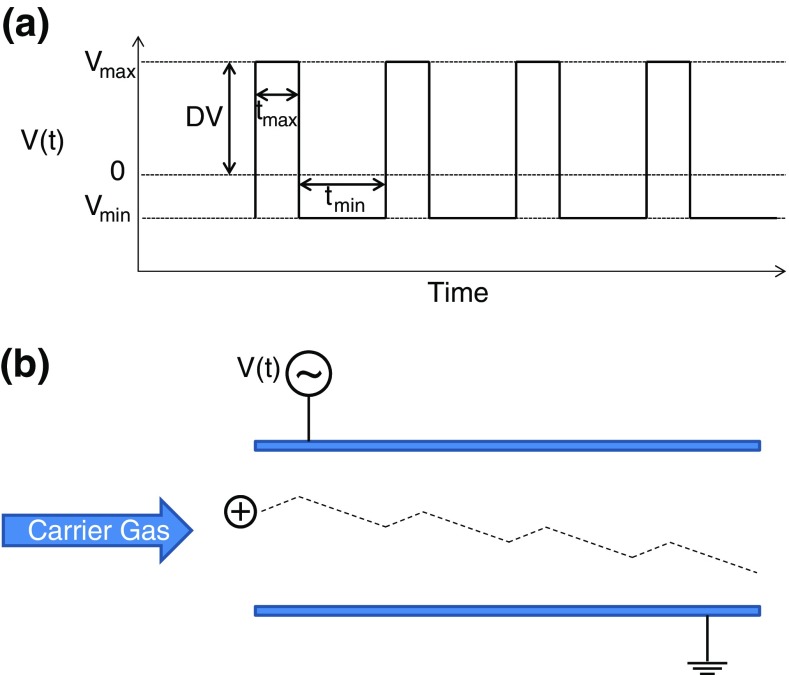
(**a**) Asymmetric waveform applied to the FAIMS electrodes; (**b**) ion trajectory through the FAIMS device

In FAIMS, ions are entrained by a carrier gas between two parallel electrodes, which can be planar or curved. The application of a high frequency waveform to one of the electrodes generates an oscillating electric field, see Figure 1. A key requirement is that the waveform is asymmetric (i.e., V_max_ ≠ -V_min)_. V_max (_i.e., V_0-P)_ is known as the *dispersion voltage*, *DV*, (and sometimes the separation voltage, SV), and the resultant electric field is the *dispersion field*, *DF*. A second requirement is that the net electric field during one cycle of the waveform is zero (i.e., E_max_.t_max_ = -E_min_.t_min)_. (Ideally, the applied waveform would be square as shown in Figure 1. In practice, the applied waveforms are generally bisinusoidal [4, 9, 10]). If an ion has identical mobility in both the high and low segments of the electric field, its net displacement from the original trajectory will be zero (ignoring effects of ion diffusion). If, however, there are differences in its mobilities in high and low fields, (see Equation 2 above), it will become displaced towards one of the electrodes. With each cycle of the waveform, this displacement will become more pronounced until eventually the ion will collide with the electrode and become neutralized. To prevent this occurrence, a DC voltage, the *compensation voltage*, *CV*, is applied to the electrode. The *compensation field*, *CF*, counteracts the displacement of the ion, thus enabling it to be transmitted through the FAIMS device. The required CF for a particular ion is dependent on its differential mobility—the difference in mobility in the high electric field segment and in the low electric field segment.

**Fig. 2:**
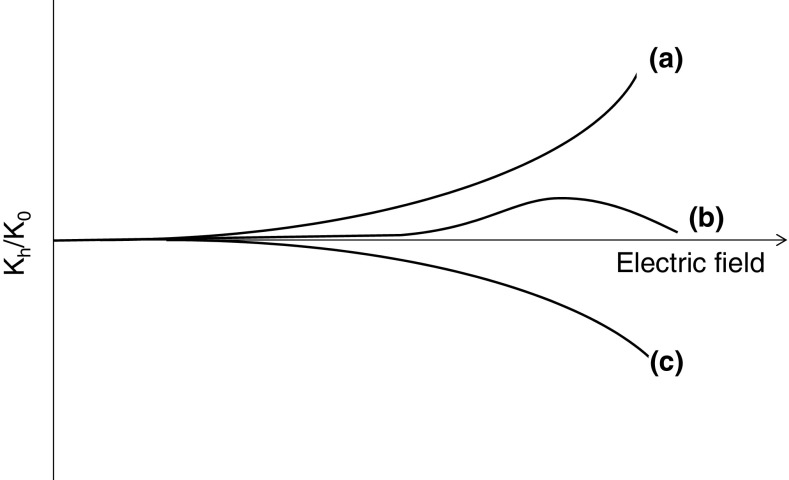
Dependence of ion mobility on electric field. Three types of behavior are observed: A-, B-, and C-type

Three types of field dependence have been described: (1) ion mobility increases with increasing electric field; (2) ion mobility initially increases and subsequently decreases with increasing field; and (3) ion mobility decreases with increasing fields. These dependences have been termed A-type, B-type, and C-type, respectively [3], see Figure 2. These terms, however, are problematic: they can only be applied relative to the range of electric field strengths being considered. If an experiment considers fields below the B-type maximum only, how to differentiate between A- and B-type? Conversely, at fields above the B-type maximum, are both B- and C-type ions classified as C-type? Moreover, all ions will eventually show decreased ion mobility with increased field [1, 11]; thus all A-type ions are really B-type ions.

## What Sets FAIMS Apart from Other Ion Mobility Spectrometry Techniques?

As mentioned above, FAIMS is quite different from conventional ion mobility spectrometry techniques, such as drift tube ion mobility spectrometry (DTIMS) and traveling wave ion mobility spectrometry (TWIMS) [12–14]. The following describes the key differences:

- In FAIMS, ions are transported through the device by a flow of gas. In conventional IMS, ions are transported by an electric field.
- In FAIMS, the electric field is applied perpendicular to the (net) direction of travel (i.e. direction of gas flow). In conventional IMS, the direction of the field and of ion movement are the same.
- In FAIMS, the electric field oscillates. In conventional IMS, it is constant. (It should be noted that in TWIMS, the electric field oscillates along the direction of travel, although the overall field along the ion mobility device is constant).
- In FAIMS, ions are separated according to electric field (compensation field) whereas in conventional IMS, ions are separated temporally. Consequently, FAIMS provides a continuous flow of ions and conventional IMS provides discrete packets of ions.
- To date, FAIMS cannot be used to directly determine the collision cross section (Ω) of an ion. In conventional ion mobility spectrometry (i.e., in the low field limit) several models exist for calculation of Ω including projection approximation (PA) [15], trajectory model (TM) [16], exact hard-sphere scattering (EHSS) [17], and projection superposition approximation (PSA) [18]. No such model exists currently for high field ion mobilities, and differential mobilities cannot be ascribed to ion structures. It is possible to hyphenate FAIMS with other techniques to measure collision cross sections. For example, Smith and co-workers have coupled FAIMS with conventional IMS [19, 20], and Purves et al. coupled an energy loss method in a triple quadrupole mass spectrometer with FAIMS [21].

FAIMS also differs from differential mobility analysis (DMA) [22]. In DMA, ions travel between two electrodes, in a direction perpendicular to a flow of gas. The gas flow thus displaces the ion. Only ions with specific mobility, determined by the voltage between the electrodes and the flow rate of the gas, are able to traverse the gap. The potential for confusion here is confounded by the fact that FAIMS is often referred to as DMS!

## Mechanisms of Separation in FAIMS

At low electric fields, ion mobility is governed by the temperature of the bath gas. At high electric fields, the mobility depends on both the temperature of the gas and the velocity of the ion through the bath gas (and therefore the strength of the field) [23]. A crucial factor is long-range ion-induced dipole attractive forces between the ion and the bath gas. The strength of these interactions depends on the polarizability of the bath gas and the size and charge of the ion and, hence, will vary with gas composition and nature of the ion. If the thermal energy of the bath gas is small relative to the potential well of the attractive force, collisions between the ion and bath gas will result in energy gains by the ion. Hence ion mobility will increase with increasing electric field strength (A-type). Conversely, if the thermal energy is similar or greater than the potential well of the attractive force, collisions will result in energy loss by the ions. That is, ion mobility will decrease with increasing electric field (C-type).

FAIMS performance (resolution, peak capacity) can be improved through use of mixtures of gases [24–30]. Blanc’s law states that the mobility of an ion in a mixture of gases relates to the mobility in the individual gases scaled by their abundance. That is, for a mixture of gas X and gas Y with relative abundances *x* and *y*, the mobility of an ion in the mixture, *K*_*XY*_, can be calculated as

3 $$ \frac{1}{K_{XY}}=\frac{x}{K_X}+\frac{y}{K_Y} $$

where *K*_*X*_ and *K*_*Y*_ are the mobilities in gas X and gas Y individually. Blanc’s law only holds when the electric field strength approaches zero. Deviations from Blanc’s law at high fields are relatively small but because FAIMS relies on *differences* in mobilities, the effects on FAIMS performance are considerable. The effects are most significant when differences in the masses of the gas components and the ion mobilities within the components are large. Shvartsburg and colleagues concluded that a binary mixture comprising He and SF_6_ would constitute the ultimate FAIMS carrier gas [31].

Eiceman and co-workers showed that the presence of water in the carrier gas affected the FAIMS spectra of organophosphorus compounds [32]. They proposed a mechanism of separation in which ion-neutral clusters form during the low field segment of the FAIMS waveform and dissociate during the high field segment. The larger collision cross section of the cluster serves to decrease ion mobility during the low-field segment while the ‘naked’ (or near naked) ion has increased ion mobility during the high-field segment. Thus, clustering/declustering amplifies the differential mobility of the ion. An obvious corollary is that addition of modifiers to the carrier gas should improve FAIMS separation capacity. First illustrated for nitro-organic explosives [33], this approach has since been more widely applied [6, 34–37]. Blagojevic et al. showed that polar modifiers can suppress proton transfer to other modifiers thereby improving sensitivity [34]. Purves et al. showed that addition of acetonitrile improved peak capacity [35]. Work by Ruotolo et al. suggested that conformational selectivity might be improved more generally in ion mobility spectrometry through judicious selection of drift gas [38]. Further support for the clustering model comes from the work of Russell and co-workers [39].

A detailed investigation by Schneider et al. [40] of a range of low molecular weight species (including small peptides) showed that FAIMS separation is a delicate balance of the clustering model and the hard-sphere scattering model. For polar carrier gases and modifiers, the clustering model dominates, and A-type behavior is observed. In the absence of polar modifiers, and particularly in helium, hard-sphere scattering dominates, and C-type behavior is observed. As mentioned above, at sufficiently high fields, mobility will decrease for all ions because of the hard repulsive core of the ion neutral interaction.

A third model has been proposed to explain the behavior of some protein ions in FAIMS [41]. Shvartsburg et al. investigated the FAIMS transmission of 10 proteins within a molecular weight range of 8 to 66 kDa [41]. They found that proteins with mass < ~30 kDa showed a decrease in mobility with increasing electric field (C-type), whereas those with mass >30 kDa showed an increase in mobility with increasing electric field (A- or B-type behavior). It was concluded that the A- (or B-) type behavior was the result of reversible dipole alignment of the high mass ions during the high electric field segment of the FAIMS waveform. That is, all large protein ions have a dipole, which will align in a sufficiently strong electric field. Once aligned, the mobility of the ion will be dictated by the collision cross section of the ion in the plane orthogonal to the dipole, rather than the rotationally-averaged collision cross section. Note that the dipole of the protein ion is not simply that of the native conformation of the protein but may be enhanced or reduced by unfolding in the electrospray process (denaturation in organic solvent and Coulomb repulsion from multiple charges). There are two points to consider relating to dipole alignment in FAIMS:

First, what is the relative orientation of the dipole to the molecular axis with the greatest magnitude (i.e., the longest dimension of the ion)? If it is parallel, the collision cross section in the orthogonal plane will be smaller than the rotationally-averaged collision cross section and the mobility will increase (A- or B-type). If the dipole is orthogonal to the long molecular axis, the reverse is true and C-type behavior will be enhanced. The latter is difficult to prove: How can ‘normal’ C-type behavior (which cannot be predicted) be distinguished from ‘enhanced’ C-type behavior?

Second, the benefits of dipole alignment in FAIMS separation (i.e., by augmenting the differential ion mobility) are only realized when alignment occurs only during the high field segment of the waveform. If the dipole is sufficiently large (or the electric field in the low-field segment sufficiently high), dipole alignment will occur throughout the entire waveform.

Shvartsburg et al. subsequently developed a model for pendular dipole alignment of ions in gases, which considered the effect of rotational heating [42]. The model reveals that for a particular electric field strength, a minimum dipole is required for alignment. Similarly, for a specific dipole, a maximum electric field for alignment exists. These limits arise because at some field strength value the rotational heating resulting from the electric field will counteract the dipole alignment. It was concluded that the minimum dipole required for alignment was ~450 D and that as dipole moment correlates approximately with protein mass, this corresponds to a protein mass of ~30 kDa. That conclusion was further corroborated in experiments performed on ubiquitin (8.6 kDa) and bovine serum albumin (66 kDa) by use of a miniaturized FAIMS device, which enables use of much higher electric fields than standard FAIMS devices [43]. FAIMS analyses of myoglobin (17 kDa) and cytochrome *c* (12 kDa) have revealed evidence for dipole alignment for particular charge states, presumably due to some of the ensemble of protein conformations having sufficiently high dipole moment [44].

## Analysis of Intact Proteins by FAIMS

FAIMS was first applied to protein ions by Purves and Guevremont [45]. They showed that the charge state distribution observed for cytochrome *c* was dependent on FAIMS conditions. In further work on ubiquitin, they showed that for some charge states, multiple conformers could be resolved [46]. They speculated “…that the position of the conformer in a CV spectrum is a function of its ability to change structure in response to the applied electric field”; however, if an ion changes structure during transit through the FAIMS device, the new structure may not be transmitted at the same compensation field (i.e., the ion will collide with electrodes as a result of its (new) differential mobility [47]). This characteristic has been termed the “self-cleaning” mechanism [48] and is an important consideration when discussing FAIMS of protein ions.

Protein ions exist as an ensemble of low-energy conformers separated by small barriers. Heating the protein ions results in unfolding and population of other conformational states. The high electric fields applied in FAIMS means that ions will be collisionally heated and, therefore, protein ions may be unfolded. This effect was first considered for ubiquitin and cytochrome *c* ions by Purves and co-workers. They showed that the increase in average temperature during one cycle of the FAIMS waveform was ~7 K for ubiquitin [26] and ~ 10 K for cytochrome **c** [47], concluding that changes in conformational structure should be minimal. Shvartsburg [48], however, argued that it is the change in *maximum* temperature rather than average temperature that is relevant, using the neat analogy of egg white protein: the egg white protein will become denatured by intermittent boiling separated by longer periods in cold water, regardless of the fact that the average temperature across the time period is <100 °C. Shvartsburg showed that the effect of FAIMS on ubiquitin ions was equivalent to heating them by ~50–55 K above room temperature (i.e., in agreement with the maximum temperature experienced in the FAIMS cycle).

What is the effect of field-heating of protein ions on FAIMS spectra? The conformational changes occur in short time periods near the waveform peaks. If the unfolding results in small changes in differential ion mobility, small shifts in compensation field may be observed; however, large changes during transit would result in the ion being lost because of the self-cleaning mechanism. If unfolding occurs instantaneously (within the first cycle of the waveform), the ion will not be filtered out, i.e., there will be only one relevant compensation field (that of the unfolded conformer) rather than two (pre- and post-unfolding). This rapid unfolding has been likened to annealing of ions by energetic injection into conventional ion mobility spectrometry [48]. Field heating effects may be advantageous (e.g., improving resolution by reducing spectral complexity [49, 50]), or detrimental (e.g., by distorting conformers intended for subsequent analysis by conventional ion mobility spectrometry), to FAIMS. In the latter case, the effect can be minimized by cooling the carrier gas [51]. Field heating is further augmented by the addition of lighter gases, such as helium, to the carrier gas [27, 28]: The increase in temperature due to field heating is inversely proportional to Ω^2^ [52], and collision cross sections are smaller in lighter gases.

**Fig. 3:**
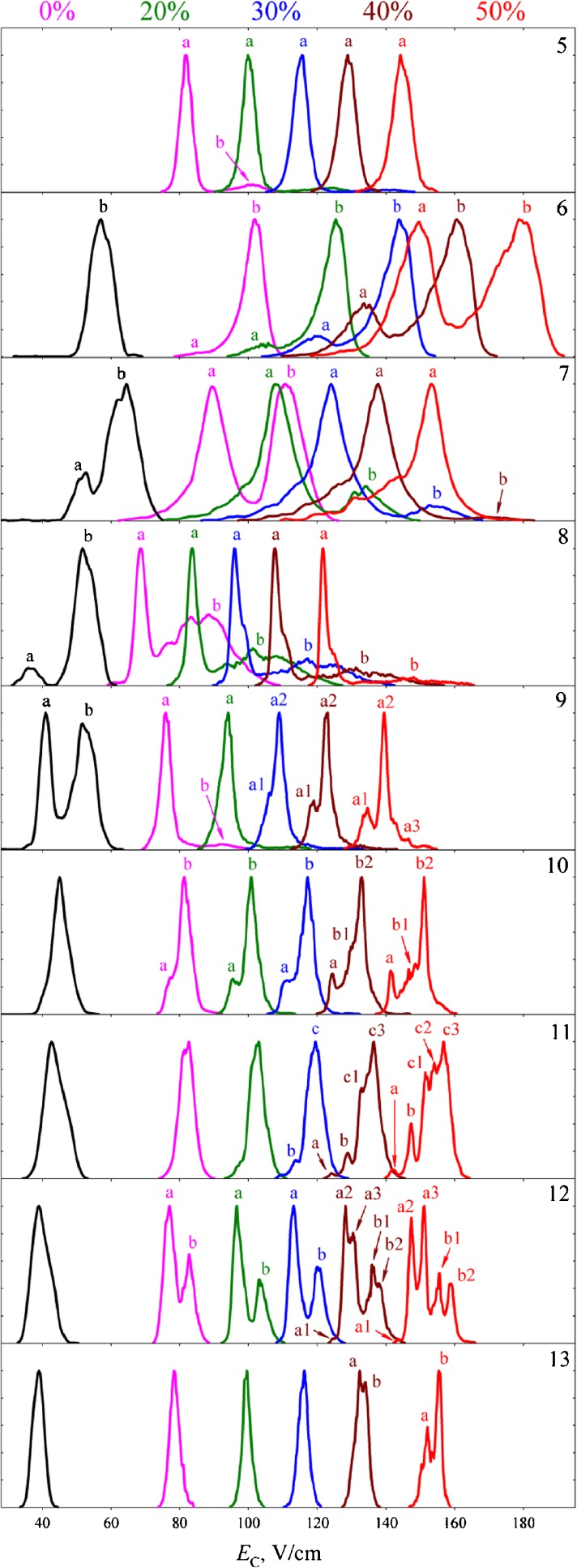
Normalized FAIMS spectra for ubiquitin ions with charge states +5 to +13 (as denoted top right of each panel) obtained at dispersion field 28 kV/cm and carrier gas He:N_2_ with % He indicated. Spectrum shown in black was obtained using a cylindrical FAIMS device at dispersion field 20 kV/cm and N_2_ carrier gas. Selected conformers are labeled. Reprinted with permission from [49]. Copyright 2012 American Chemical Society

**Fig. 4:**
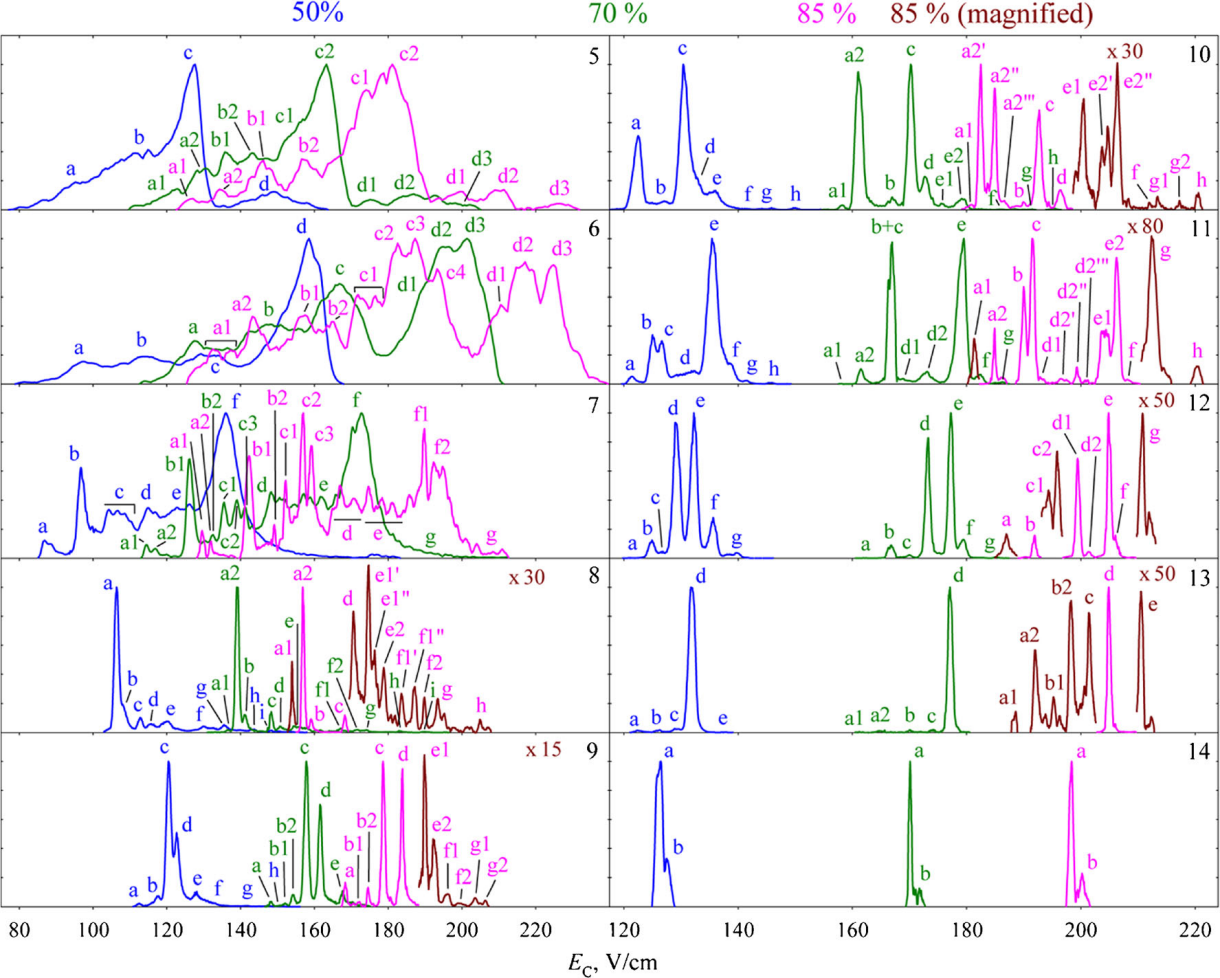
FAIMS spectra for ubiquitin ions with charge states +5 to +14 (as denoted top right of each panel) obtained with carrier gas H_2_:N_2_ with % H_2_ indicated. Smaller features are magnified for 85% H_2_ only. Reprinted with permission from [50]. Copyright 2013 American Chemical Society

Not surprisingly, much of the work on FAIMS separation of protein conformers has focused on ubiquitin and cytochrome *c* [19, 21, 26, 46, 47, 53, 54]. The gas-phase conformers of these proteins are very well characterized (for example, see [55–63]). FAIMS has been combined with hydrogen deuterium exchange [47, 53], with conventional ion mobility spectrometry [19], and with electron capture dissociation mass spectrometry [54]. FAIMS has also been combined with cold ion spectroscopy for the investigation of conformers of the peptide bradykinin in elegant experiments by Papadopoulos et al. [64]. Purves et al. showed FAIMS separation of four conformers of the 11+ and 12+ of ubiquitin using nitrogen as the carrier gas [26]. Shvartsburg and Smith showed that by using a combination of higher dispersion field and a carrier gas comprising up to 50% He, it was possible to separate four major conformers of the +12 charge state of ubiquitin [49] with a resolving power of ~80, see Figure 3. Replacing helium with hydrogen allowed the separation of over 10 conformers for each of the charge states of ubiquitin [50], see Figure 4. Similar improvements in resolution were observed for conformers of cytochrome *c* and myoglobin [44]. These results have been rationalized as follows [50]: the peaks observed in conventional ion mobility spectrometry or FAIMS at lower dispersion fields and absence of He or H_2_ correspond to ensembles of conformationally similar protein ions. On field heating (either by increasing DF or addition of lighter gases), these families of conformers are annealed to yield a single protein conformer, in addition to “self-cleaning” of other conformers.

One might reasonably ask what the relevance of FAIMS separated protein conformers have to nature, where the structure of a protein is inextricably linked to its function? In particular, what is the significance of the conformers revealed by enhanced field-heating? In the absence of enhanced field heating, it is possible to correlate FAIMS separated conformers with those observed in conventional ion mobility spectrometry [19]. It is also possible to transmit noncovalent protein complexes (dimer of bovine serum albumin) through a FAIMS device [42]. It may be that FAIMS can provide information on native conformations. Indeed, FAIMS has been applied to the analysis of conformers of the amyloidogenic protein β_2_-microglobulin and an acid destabilized variant, yielding results consistent with circular dichroism spectroscopy and NMR [65]. In terms of the FAIMS field-heating annealing of protein conformers, the likely benefits are in the separation of isomers or isobars (e.g., the separation of post-translationally modified proteins or substitution variants, thus aiding top-down proteomics).

**Fig. 5:**
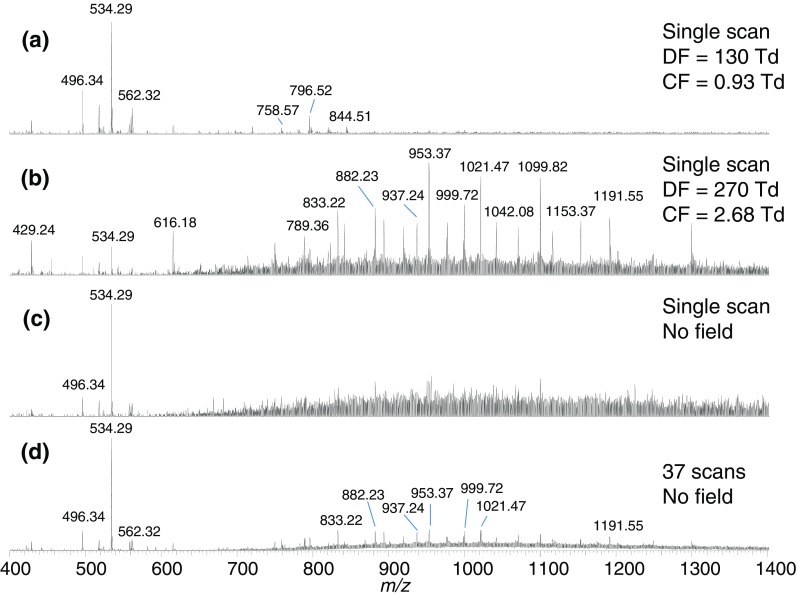
LESA 2D-FAIMS mass spectrometry of mouse liver. (**a**) Single scan mass spectrum at DF = 130 Td, CF = 0.93 Td; (**b**) single scan mass spectrum at DF = 270 Td, CF = 2.68 Td; (**c**) single scan mass spectrum recorded in the absence of FAIMS field; (**d**) mass spectrum recorded in the absence of FAIMS field comprising 37 co-added scans (∼1 min data). Reprinted from [66]

**Fig. 6:**
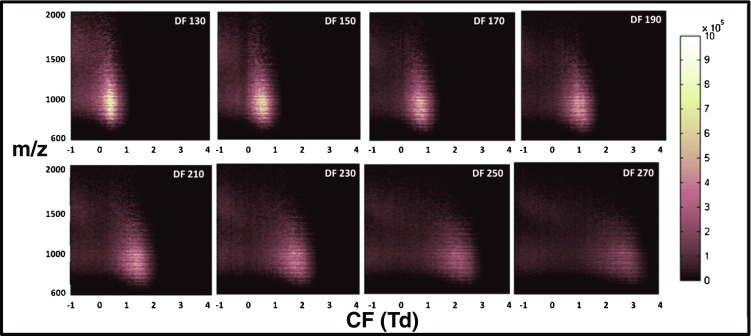
Total ion transmission maps obtained following LESA 2D FAIMS analyses of mouse liver. Reprinted from [66]

It would be short-sighted to consider the merits of FAIMS solely in relation to its ability to separate conformers of a particular, and individual, protein. As a separation technique, it clearly has potential benefits for the analysis of complex mixtures of proteins such as those extracted from biological substrates. This feature has recently been exploited in our laboratory by coupling FAIMS with the ambient technique of liquid extraction surface analysis (LESA) for the analysis of thin tissue sections, bacterial colonies growing on agar, and dried blood spots [66, 67]. This work employed an ultrahigh field miniaturized FAIMS device. The incorporation of FAIMS resulted in increased signal-to-noise and information-rich protein mass spectra. In addition, proteins and lipids could be separated. These advantages are illustrated in Figure 5, which shows mass spectra obtained from a two-dimensional (DF and CF) FAIMS analysis of LESA-sampled mouse liver tissue compared with LESA mass spectra obtained in the absence of FAIMS. A key feature of the LESA 2D FAIMS analysis is the separation of lower molecular weight (<30 kDa) and unresolved higher molecular weight proteins, presumably as a result of the dipole alignment mechanism. This separation can be visualized in the total ion transmission maps shown in Figure 6. This application area sees the translation of FAIMS into the true biological arena and, as such, is particularly promising.

## Bottom-Up Protein Analysis

Protein analysis can also be considered from the bottom-up perspective, that is, the analysis of proteolytic peptides. Peptides were first analyzed by FAIMS by Guevremont and Purves [45, 68]. They showed that small peptides exhibit A-type behavior, whereas larger peptides are C-type. Building on that work, they analyzed a tryptic digest of porcine hemoglobin [69]. Their results showed that FAIMS reduced the chemical background, thus increasing signal-to-noise ratios. This observation is attributed to the differing optimum CFs for transmission of the background ions and the peptide ions and/or the background ions dissociating during the FAIMS separation with the fragments having a different optimum CF to the parent ion (the self-cleaning mechanism). Additionally, they showed that isobars (a singly charged ion and a doubly charged ion, both of *m*/*z* 532) could be separated [69]. These researchers subsequently coupled FAIMS with MS/MS for the analysis of tryptic peptides [70]. The separation of chemical background from peptide ions resulted in simplified MS/MS spectra, which were more straightforward to interpret. A more extensive study followed, in which tryptic peptides from a range of proteins and mixtures of proteins were analyzed [71]. That study led the way for FAIMS proteomics because it considered theoretically how FAIMS might be coupled with liquid chromatography (LC), specifically the challenge of integrating the discrete packets of analytes produced by LC and the time spent scanning CF in the FAIMS device. They concluded that the most suitable approach for an LC FAIMS MS/MS experiment would be a “stepping” approach in which rather than scanning the full range of CFs, a number of discrete CF values are sampled.

**Fig. 7:**
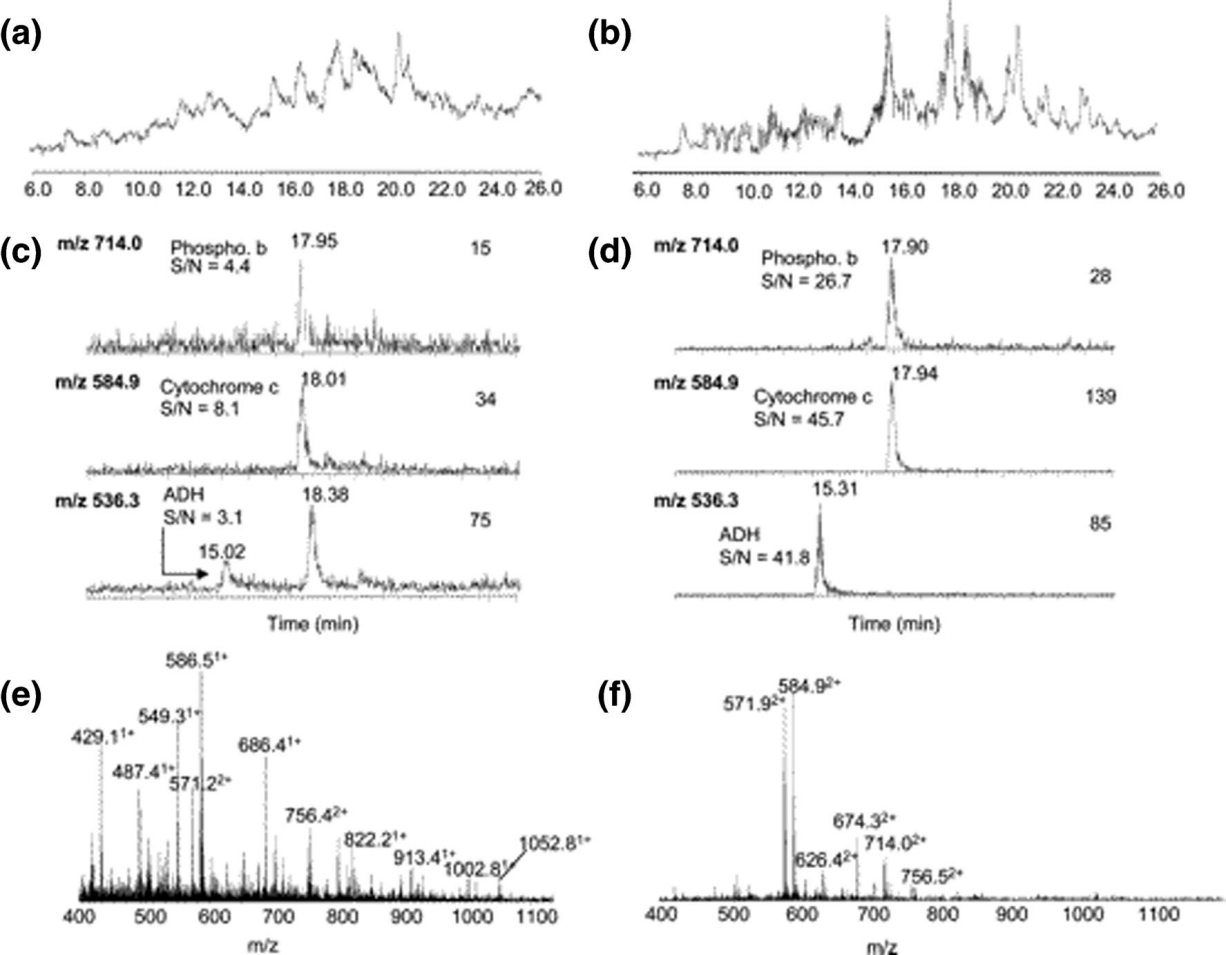
Nano-liquid chromatography mass spectrometry analysis of a five protein digest (20 fmol each) (**a**) without and (**b**) with FAIMS. Extracted ion chromatograms of the doubly-charged ions at *m*/*z* 714.0, 584.9, and 536.3 (**c**) without and (**d**) with FAIMS. Extracted mass spectra for peak eluting at ~18 min from the nano-LC mass spectrometry analysis (**e**) without and (**f**) with FAIMS. Reprinted with permission from [72]. Copyright 2005 American Chemical Society

FAIMS was first coupled with LC MS/MS for proteomics analyses on a Q-TOF mass spectrometer by Thibault and co-workers [72]. A stepping method was employed in which three discrete CV values were sampled during the acquisition. (In a later publication [73], we have designated this approach the ‘internal stepping’ method as the CV is stepped within a single LC analysis. In contrast, the ‘external stepping’ method refers to multiple LC MS/MS analyses, each at a separate, and constant, CV). Thibault and co-workers showed that FAIMS afforded improvements in signal-to-noise ratios of up to 12-fold because of its ability to separate (largely singly charged) chemical background from multiply charged tryptic peptides, as illustrated in Figure 7. MacCoss and co-workers [74] applied LC FAIMS MS/MS to the analysis of a tryptic digest of proteins from *Saccharomyces cerevisiae* on an ion trap instrument. They used an internal stepping method comprising five CV values and showed that the peak capacity of the experiment was comparable to a that of a multi-dimensional protein identification technology (MudPIT) experiment but with the benefit of increased throughput (analysis time of 90 min (FAIMS) versus 24 h (MudPIT)). The work made use of traverse cylindrical FAIMS electrodes (the Thermo FAIMS device) and the results revealed a drop in absolute signal intensity (i.e., the transmission efficiency through the device was ~10%. (Originally attributed to lack of lateral confinement of the ions, later work by Prasad et al. showed that the poor transmission efficiency of this device was due to a combination of angular desolvation gas flow directing ions away from the entrance to the FAIMS device and the FAIMS carrier gas directing ions entering the device onto the inner electrode [75]). Despite this limitation, the reduction in interference from chemical background meant that the dynamic range was observed to increase by 5-fold. Saba et al. [76] coupled LC FAIMS with an Orbitrap mass spectrometer, again using the internal stepping method. They compared results obtained with and without FAIMS for the analysis of tryptic digest of simple protein mixtures and whole cell lysates from human U937 cells. Inclusion of FAIMS resulted in a 55% increase in the number of assigned MS/MS spectra from 1958 unique identifications without FAIMS to 3034 with FAIMS. The improved S/N observed with FAIMS allowed the threshold intensity for MS/MS fragmentation to be lowered in these experiments and subsequent identification of over 450 low abundance peptides. Similar results were obtained when LC FAIMS MS/MS was applied to the analysis of the *Drosophila melanogaster* phosphoproteome [77]. A total of 2168 phosphopeptides were identified without FAIMS compared with 3476 with FAIMS, a 51% increase. Swearingen et al. [78] applied an external CV stepping method to the analysis of stable amino acid in cell culture (SILAC)-labeled digests of *Saccharomyces cerevisiae*. The traverse cylindrical FAIMS device was used in combination with a modified electrospray source and sheath gas, which improved the transmission efficiency from ~3% to ~17% for 2+ ions of angiotensin. Nevertheless, they observed an increase of 50% in peptide identifications and 64% in protein identifications. In 2013, our laboratory published a comparison of the internal and external CV stepping methods, and a proteomics workflow incorporating strong cation exchange chromatography in the analysis of whole cell lysates from a breast carcinoma cell line [73]. Transmission efficiency was 10%–20%. We found that the external CV stepping method resulted in a greater number of protein identifications than the internal CV stepping method. This observation is due to the longer duty cycle of the internal stepping method. That is, for a specific CV, the method will cycle through a number of other CV values before returning to the original CV, and for many peptides the chromatographic peak will not coincide with the optimum CV and the subsequent mass spectrum will have reduced quality. The external stepping approach is inherently more expensive than the internal stepping approach in terms of both analysis time and sample, and in situations where sample is limited or high-throughput is required, the internal stepping method may prove to be more appropriate. In addition, we found that the identifications arising from the FAIMS workflow were complementary to those obtained via the SCX workflow. Ergo, for maximum proteome coverage both LC FAIMS MS/MS and (2D) LC MS/MS should be employed. That approach was applied in a recent phosphoproteomics analysis of the effects of chemical inhibition of fibroblast growth factor receptor signaling in breast cancer carcinoma cells [79]. The combined number of high confidence phosphorylation sites identified was 2538, 685 of which were novel to the FAIMS dataset. The FAIMS dataset contained a greater proportion of phosphothreonine and phosphotyrosine sites, and importantly an increased number of multiply-phosphorylated peptides, a class of species typically overlooked in standard LC MS/MS proteomics analyses.

An area in which FAIMS has potential to play a considerable role in the bottom-up analysis of proteins is that of isomer separation. Isomer separation by FAIMS was first demonstrated for amino acids leucine and isoleucine by Barnett et al. [80] More recently, Mie et al. have shown the separation of enantiomers of amino acids by addition of a chiral reference compound and FAIMS analysis of the resulting diastereomeric complexes [81]. As well as enantiomers and diastereoisomers, in proteomics studies, isomers can arise through sequence inversions, that is, those in which the order of amino acid residues are altered, and localization variants of post-translationally modified peptides (i.e., peptides with identical sequence but differing sites of modification). Separation of the latter by FAIMS was first demonstrated by Xuan et al. for phosphopeptides with sequence APLSFRGSLPKSYVK in which the serine residues are variously modified [82]. In that work, the traverse cylindrical FAIMS device was employed. Improved separation of those phosphopeptides was achieved by use of planar electrodes and helium-rich carrier gas [83]. FAIMS separation of phosphopeptide isomers with adjacent modification sites and both serine and threonine phosphorylation was subsequently demonstrated [84]. Localization variants involving other modifications can also be separated by FAIMS, including glycosylation [85], acetylation [86], and methylation [87]. The field is now building on these initial proof-of-principle investigations and starting to exploit this benefit in addressing real biological challenges. Bridon et al. identified several isomeric phosphopeptides in their FAIMS phosphoproteomics study of insulin signaling in *Drosophila melanogaster* [77]. Ulasi et al. employed LC FAIMS MS/MS to obtain a comprehensive map of glycosylation of the flagellin protein from *Campylobacter jejuni* [88]. Zhao et al. have applied LC FAIMS MS/MS to study phosphorylation in human breast carcinoma cells [79].

FAIMS separation of peptide sequence inversions has also been demonstrated. Initially this was shown for nitrated peptides with sequence A_x_nYA_y_K (where x = 0–6; y = 6–0; nY is nitrotyrosine) by use of a planar FAIMS device and helium-rich carrier gas [89]. A comprehensive study of this phenomenon considered a phosphopeptide library comprising the sequences GPSGXVpSXAQLX(K/R) and SXPFKXpSPLXFG(K/R), where X = ADEFGLSTVY (i.e., total number of library members = 4000, but total number of unique peptide masses = 556 because of the presence of isomers) [90]. Analysis of the peptide library by reversed-phase (RP) LC MS/MS resulted in identification of 8% of the library; by SCX RPLC MS/MS gave 17% and by RPLC FAIMS MS/MS gave 35%. The maximum number of isomers identified for a single peptide mass by RPLC FAIMS MS/MS was 12 and for SCX RPLC MS/MS were 7 out of a possible 18. That work demonstrates an advantage of FAIMS more generally for proteomics. The separation of co-eluting species by FAIMS affords cleaner mass spectra and consequently higher scoring peptide identifications.

FAIMS has also found applications in targeted analysis of peptides. Although these studies are not strictly protein analysis, they are described in brief here. Ells et al. [91] showed that FAIMS mass spectrometry could be successfully applied to the analysis of microcystins—peptide hepatotoxins derived from cyanobacteria—with lower limits of detection than ESI mass spectrometry. Jemal and co-workers [92] and Roemer and co-workers [93] both developed an LC-FAIMS-selected reaction monitoring (SRM) assay for quantifying proprietary peptide drug candidates in plasma and serum. Bailly-Chouribery et al. [94] applied the FAIMS-SRM approach to the analysis of tryptic peptides of recombinant human erythropoietin, a glycoprotein used in horse doping. Most recently FAIMS has been applied to the detection to peptide antigens from a leukaemia cell line [95].

## Summary

To answer the question posed by this article’s title – “*To what extent is FAIMS beneficial in the analysis of proteins*?” – FAIMS has the potential to be highly beneficial and this potential is starting to be realized. At present, there are three FAIMS devices commercially available: the Thermo Fisher cylindrical device, the Sciex planar device, and the Owlstone miniaturized planar device. FAIMS cannot currently be used for the direct calculation of collision-cross section but that role is fulfilled by conventional ion mobility spectrometry. The true value of FAIMS comes from its role as a separation device. For example, FAIMS is capable of separating protein conformers, which has potential benefits for top-down proteomics and structural biology. The latter is offered with the caveat that there remains a knowledge gap concerning field-heating and observed conformers. An exciting application of FAIMS is the separation of complex mixtures of proteins extracted from biological substrates, thus enabling direct surface sampling and potentially imaging of previously undetected proteins from those substrates. From a bottom-up perspective, again the advantage of FAIMS lies in its separation capabilities. FAIMS has been coupled with LC MS/MS for proteomics analyses. Several groups have shown that LC FAIMS MS/MS is complementary to the LC MS/MS approach and thus improves proteome coverage. Further benefits may derive from optimization of carrier gas and gas modifiers. In the drive towards total proteome coverage, FAIMS will provide an invaluable tool. The ability of FAIMS to separate post-translationally modified peptide isomers is particularly useful and the field is starting to move towards applying FAIMS to real world challenges.
